# Assessment of Shoulder Function after Internal Fixation of Humeral Diaphyseal Fractures in Young Adults: A Prospective Comparative Study

**DOI:** 10.1155/2021/9471009

**Published:** 2021-11-01

**Authors:** Hossam Fathi Mahmoud, Ahmed Hatem Farhan, Fahmy Samir Fahmy

**Affiliations:** Orthopedic Surgery Department, Faculty of Medicine, Zagazig University, Zagazig, Egypt

## Abstract

**Background:**

Humeral shaft fractures are commonly encountered in casualties. There are different methods of operative internal fixation with no consensus on the best technique. The objective of this study was to assess shoulder function and rate of complications among two different options of fixation, intramedullary nailing, and minimal invasive plate osteosynthesis (MIPO) in young adults.

**Methods:**

Forty-two patients with humeral shaft fractures were included in the study and divided into two equal groups: group A treated with antegrade intramedullary locked nails (IMN) and group B with MIPO. Fracture union was evaluated with serial X-rays, and shoulder function was assessed in both groups using the scale of the American Shoulder and Elbow Surgeons (ASES), University of California at Los Angeles Shoulder Scale (UCLA), and visual analog score (VAS). The mean differences between groups were recorded and considered significant if the *P* value was ˂0.05.

**Results:**

The results were reported prospectively with no significant differences in mean age, sex, side of injury, type of fracture, mechanism of injury, and the follow-up period between the groups studied. Group A had shorter operative time and minimal blood loss than group B. Regarding shoulder function scores (ASES, UCLA, and VAS), the results in the MIPO group were better than the IMN group with shorter time of union and fewer complications.

**Conclusion:**

Despite a shorter operative time and lower blood loss during locked intramedullary nail fixation in the management of humeral shaft fractures, MIPO enables more superior shoulder function with better fracture healing and lower morbidities.

## 1. Introduction

Humeral shaft fractures represent 3% of all adult fractures. Conservative management remains the main stay for treatment of stable and nondisplaced fractures, but in certain conditions, surgical intervention is needed [1–4].

Poor compliance with conservative treatment and failure to maintain reduction, as well as open fractures, segmental fractures, neurovascular insults, floating elbows, and polytrauma patients with multiple fractures are the main indications for surgical intervention [5].

Advances in the last few decades in the design and manufacture of modern surgical implants used for fixations have helped expand the indications for operative intervention with the achievement of early fracture union and lower complications [6].

The choice of the ideal method of fixation for humeral shaft fractures remains controversial, and there is no consensus in literature about the best method. However, both locked intramedullary nail (IMN) and minimal invasive plate osteosynthesis (MIPO) are accepted surgical options that enable minimal invasive biological fracture fixation [5].

Although open dynamic compression plating (ORIF) provides more accurate anatomical reduction and rigid fixation and reduces the risks of malunions, it requires wide intraoperative exposure with more soft tissue injury. This may contribute to high infection rates and increased rates of nonunion due to violation of soft tissue at the fracture site and severance of periosteal blood supply [7].

The use of intramedullary locked nails is considered more superior to (ORIF) for fixation of humeral diaphyseal fractures because it is minimally invasive with less soft tissue stripping and results in less infection rates and rapid return to activities. The shoulder problems at the insertion site of the nail are the main concern of this fixation method, which can potentially be avoided with modern straight nail designs. Accidental injury to the rotator cuff, shoulder impingement, and accumulation of debris from reaming are the main causes of shoulder dysfunction [8, 9].

Minimal invasive plating osteosynthesis has gained popularity for the treatment of diaphyseal humeral fractures since it provides stable fixation with micromotion at the fracture site and stimulation of callus formation. It has a diminished risk of nonunion, infection, and shoulder disabilities [10].

The purpose of the current study was to assess and compare the outcomes of shoulder functions in two groups of young adult patients with humeral diaphyseal fractures treated by locked IMN and MIPO. Also, the benefits and shortcomings of each treatment method were evaluated.

## 2. Patients and Methods

Forty-two skeletally mature patients with closed humeral diaphyseal fractures were enrolled in this prospective cohort study. It was conducted at Zagazig University Hospitals between March 2017 and January 2021. 21 patients were managed with antegrade interlocking nail (group A) and 21 patients with MIPO (group B).

This work was conducted in accordance with the World Medical Association (Declaration of Helsinki) guidelines for studies involving humans. Informed consent from the patients and IRB approval from our ethical committee (ZU-IRB #65760/8-1-2017) were obtained prior to prospective collection of patient data.

Patients included in this comparative study were older than eighteen years of age and had closed humeral shaft fractures. Patients older than 50 and those who had fractures with articular extension, associated vascular injuries, floating elbows, delayed cases of more than three weeks, pathological fractures, open fractures, presence of radial nerve palsy, and distal level were all excluded from this study. The demographic criteria of the patients who participated in this study are listed in Table 1.

The patients were clinically assessed for soft tissue injury, integrity of radial nerves, vascular integrity, and presence of other fractures. Anteroposterior and lateral view X-ray films including shoulder and the elbow joints were requested for the injured limb to assess the fracture. Also, radiological evaluation of any other suspected injuries including the skull, neck, chest, pelvis, spine, and other limb injuries were done in polytrauma cases.

After primary management in the emergency room and splinting of the affected limb, the patients were admitted and prepared for surgery.

All patients were operated under general anesthesia by the same surgeon. A prophylactic antibiotic of 1 gm intravenous ceftriaxone was administered 30 minutes before surgery. Draping of the affected limb was done, and the arm was left free to help manipulation and reduction. All surgeries were done under the control of a C-arm image intensifier.

### 2.1. Antegrade Nailing Group

Patients were placed in a beach chair position. The site of entry was made through a small stab incision approximately 1 cm in length in front of the anterior rim of the acromion between the anterior and middle deltoid fibers with careful dissection down to the entry point, which was lateral to the articular margin and just medial to the greater tuberosity. The awl was placed over this point and verified with the C-arm to confirm its alignment with the medullary canal in the anteroposterior and lateral views. The medullary canal was opened with subsequent passage of the guide rod in the canal. The fracture was manipulated with gentle traction for reduction and passage of the guide through the distal part of the bone.

Prior to insertion, the guide wire whole length was determined, and the outer part of the wire was measured and subtracted from the whole length of the guide to assess the anticipated nail length.

Using a protective sleeve, reaming was started with sharp end-cutting reamer over the guide rod with 0.5 mm increments until the best fit diameter was reached. The ball tip wire was exchanged, and the nail was advanced through the medulla. The guide rod was removed after the nail had reached the distal end of the canal; 1 cm proximal to the olecranon fossa. The nail tip needs to be sunken 2 mm below the articular cartilage to avoid impingement.

After closing the fracture gap, proximal and distal locking screws were inserted under C-arm guidance, and subsequently the wound was closed in layers.

### 2.2. Plate Group (MIPO)

Patients were placed in a supine position with their arms abducted to 90° and their forearms supinated. The C-arm was placed on the same side as the limb to be operated.

A three-centimeter-long proximal incision was made between the medial border of the deltoid and biceps muscle six centimeters distal to the acromion and dissected to the humerus. The distal incision was made along the lateral border of biceps three centimeters long just proximal to the flexion crease by five centimeters. The distal incision should be far distal to the fracture site. The biceps muscle was retracted medially to identify and protect the musculocutaneous nerve, which lies above the brachialis muscle. Then, dissection was done through brachialis retracting the musculocutaneous nerve medially, while the radial nerve was protected by the lateral half of the brachialis muscle.

An extraperiosteal tunnel was made under the brachialis muscle using a periosteal elevator from distal to proximal under brachialis muscle. A plate of suitable length was passed through the tunnel and anchored to the bone with a proximal and distal screw after reducing the fracture by gentle manual traction. After proper alignment and reduction were confirmed by image intensifier, the rest of the locked screws were inserted sequentially, and the wound was closed.

**Figure 1:**
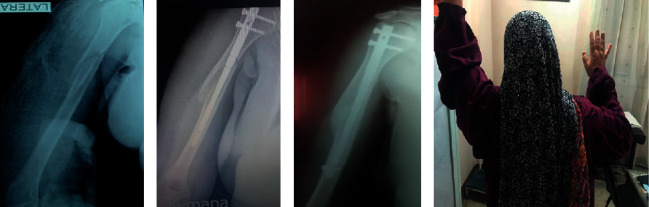
A 48-year-old female had right humeral shaft fracture AO/OTA type 12-A_2_ as shown in the preoperative X-ray (a). She was treated by interlocking nail (b). 8 months after surgery, there was complete bone union (c), but there was limited range of shoulder motion due to impingement and secondary frozen shoulder (d).

**Figure 2:**
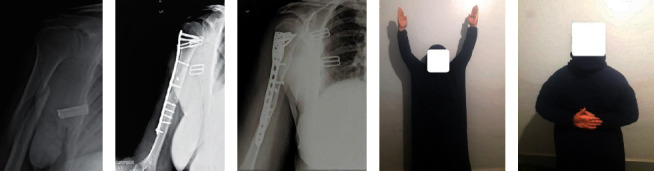
A 41-year-old female patient had humeral diaphyseal fracture due to a car accident. Preoperative X-ray shows AO/OTA type 12-B_1_ (a). She was treated by MIPO technique with complete fracture union as shown in 5 months postoperative X-ray (b, c). The patient had full shoulder function and complete range of motion (d, e).

The arm was immobilized in a sling, and antibiotic was continued for 48 hours only after surgery. Passive mobilization of the wrist, elbow, and shoulder was allowed immediately as much as could be tolerated. The stitches were removed after 14 days. Active resistance exercises were not possible until the fracture healed. Serial radiological follow-up was conducted with a monthly X-ray film until evidence of union (Figures 1 and 2). Shoulder functions were evaluated using the American Shoulder and Elbow Surgeons (ASES) score [11], University of California at Los Angeles shoulder scale (UCLA) [12], and visual analog score (VAS) [13].

### 2.3. Statistical Analysis

The data were analyzed using Statistical Package for Social Sciences software program version 16. The numerical values were recorded as means and standard deviation. Before comparing the means, the Kolmogorov–Smirnov test was used to check the normality of the groups analyzed. The means of quantitative variables were compared using independent *t*-tests, while the nominal and categorical data were compared by Chi-square and Fisher's exact tests. In all tests, *P* values below 0.05 were considered statistically significant. The sample size that gives 80% statistical power, *α* error of 0.05, and large effect size more than 0.5 was calculated using G-power software calculator version 3.1.

## 3. Results

No significant differences were identified among the two groups with respect to mean age, sex, mechanism of fracture, the affected side, type of fracture according to AO classification, time before operative intervention, and the follow-up time (Table 1).

The mean operative time showed significant differences between the two groups (*P* value <0.001), and it was shorter in the IMN group than the MIPO group by 36 minutes. Also, blood loss was less in the nail group compared with MIPO, the difference between the two groups being significant (*P*=0.035). The IMN group exhibited better results with respect to blood loss and operative time. According to the Radiological Union Scale, radiographic union occurs when a bridging callus with invisible fracture line (score 3) is seen in at least three of four cortices. The mean time of fracture healing was shorter in the MIPO group than the IMN group (12.76 ± 3.7 and 15.48 ± 4.3 weeks, respectively). The difference between the groups was significant. Regarding the results of shoulder functions, the last follow-up records of ASES, UCLA, and VAS were better in the MIPO than the IMN group with statistically significant differences (Table 2).

The overall complication rate was higher in the IMN group (23.8%) than the MIPO group (9.5%). There were five complications in group A (IMN); two cases of fracture nonunion were treated using augmentation plates and bone grafting, and three cases with shoulder pain (two patients with subacromial bursitis and one patient with partial cuff tear) were treated by shoulder arthroscopy (Figure 1(d)). There were no reported cases of nonunion and shoulder problems in the MIPO group. However, in the MIPO group, there were two cases of malunions (5°) without functional deficit. There were no recorded cases of infections and iatrogenic radial nerve palsy in both groups.

## 4. Discussion

Open reduction and internal fixation (ORIF) technique is the gold standard and most widely used operative method for treating humeral diaphyseal fractures. The major drawbacks of this technique are the need for a big skin incision, soft tissue disruption, and periosteal stripping that may predispose to higher rates of infection, radial nerve palsy, and nonunion [14].

With advances in surgical techniques, implants, and the emerging concept of biological minimal invasive fixation, both intramedullary locked nail and MIPO fixation, are commonly used nowadays for treating shaft fractures. Until now, there are controversies in literature regarding functional outcomes following both techniques and which of them is more superior [15].

This study was conducted keeping in mind the limited comparative studies between IMN and MIPO and focused on comparing the results of shoulder functions and the complication rate of both techniques among active young patients.

MIPO technique was developed to avoid soft tissue and periosteal violations associated with ORIF, so it has lower rate of postoperative infections and fracture nonunion and better cosmesis. However, most published studies have reported more operative time and blood loss for MIPO compared with IMN [16].

This is consistent with the results of our study. We found that the nail group had lower duration of surgery (88.1 vs. 124.05 minutes, respectively) and less blood loss (84.29 vs. 134.05 cc) than the MIPO group, which were statistically significant. This can be attributed to the time taken for fracture manipulation to achieve good reduction and proper alignment. Also, in a retrospective study, Wang et al. [16] showed that the nail group had shorter duration of operation with less exposure to radiation.

In a meta-analysis by Wen et al. [17], it was reported that the IMN had superior results in terms of postoperative infections than when plates were used. Contrary to that in our study, we had no cases of infection for both treatment groups. Also, they stated that the MIPO had better union than IMN and no significant difference in occurrence of iatrogenic radial nerve lesion, which is similar to what we found. These conclusions are different from the study of Wang et al. [16] who reported more nonunion and radial nerve lesions in the MIPO group.

The complication of malunion with the MIPO technique was more in the reviews published due to the indirect reduction methods used. We found two cases of malunion (varus deformity of five degrees) in the MIPO group, but this deformity did not affect the function of limb, and the patients were able to return to their normal activities. No malunion was found in the nail group. Also, Wang et al. [16] reported 2 cases of malunion in 30 patients treated by MIPO.

In the current published literature, we found contrasting results regarding nonunion for both IMN and MIPO. Wang et al. [16] had shorter time of union in the nail group, while Ma et al. [18] reported no significant difference in the nonunion rate between the nail and plate. In a cross-sectional descriptive study, Kivi et al. [19] noticed that the intramedullary nail of humeral shaft fracture fixation had a high nonunion rate. Wen et al. [17] stated that the MIPO technique had better results of union than the nail group, and this was consistent to our findings.

We report lower complication rates in the MIPO group than in the IMN, and the difference between both was statistically significant. Similar to our records, Wen et al. [17] stated that MIPO is superior to the locked nail in terms of overall complication rate.

At the end of the follow-up period, the mean VAS for the MIPO group was lower than that for IMN. This may be explained by the presence of shoulder problems and nonunion in the IMN group. Other studies have reported no significant differences for both groups [16].

We assessed the shoulder function between the nail and MIPO groups using ASES and UCLA scores and found superior functional outcomes in the MIPO than the nail group, and the difference was significant. Many published studies have not shown significant differences between both methods; however, most of them compare the IMN and ORIF techniques [16, 20–22].

The main causes of shoulder pain after antegrade IMN fixation are impingement, rotator cuff injuries, and adhesive capsulitis. We encountered three (14.2%) shoulder problems in the IMN group: two cases of subacromial bursitis were treated by arthroscopic bursectomy and debridement of the accumulated debris with rotator interval release, and the other case was a partial rotator cuff tear treated by arthroscopic repair. There were no shoulder functional deficits in the MIPO group.

In a study by Kassem et al. [23], they had two cases of shoulder impingement and limited range of motion in the nail group, which were treated by removal of the nail with no residual dysfunction. Also, Bisaccia et al. [24] described similar findings. Ouyang et al. [5] and Wang et al. [16] noticed that shoulder complications were fewer in plating, but Wen et al. [17] declared no significant difference between IMN and MIPO.

Mocini et al. [9] used an antegrade straight interlocking nail with medial entry point for fixation in their series and did not find major complications related to the nail insertion site.

All the patients in our MIPO group regained full shoulder function with satisfactory outcomes and no deficits. The rate of complications was lower than the IMN group. Davies et al. [25] compared the results of MIPO and nail groups in 30 patients, and they recommended the MIPO technique as it had less complications and better functional results.

Our study has some limitations. The sample size in our study was small, and in future studies, a larger number of patients will be needed in order to increase the confidence in the final conclusions. Also, a longer follow-up will help identify remote complications that may not appear earlier. Finally, the lack of randomization is also another weak point of this clinical study.

## 5. Conclusion

In conclusion, both MIPO and locked intramedullary nail are biological and effective techniques for the management of diaphyseal fractures of the humerus. However, locked IMN has shorter operative time, less bleeding, and less exposure to radiation, and MIPO on the other hand results in better shoulder function and union rate with lower complications. There are no differences between both techniques with respect to infection rates and radial nerve injury. Further long-term studies are recommended to confirm superiority of either technique.

## Figures and Tables

**Table 1:** The demographic and intraoperative data of the groups studied.

		IMN (group A) (*N* = 21)	MIPO (group B) (*N* = 21)	*P* value
Mean age (years)		34.8 ± 8.4	38.5 ± 8.4	0.167
Sex	Male	16 (76.2%)	15 (71.5%)	0.725
	Female	5 (28.8%)	6 (28.5%)	
Side	Right	7 (33.3%)	9 (42.9%)	0.525
	Left	14 (66.7%)	12 (57.1%)	
Mechanism of trauma	RTA	11 (52.4%)	13 (61.9%)	0.532884
	Falling	10 (47.6%)	8 (38.1%)	
AO/OTA classification	Type A	9 (42.9%)	12 (57.1%)	0.644497
	Type B	9 (42.9%)	7 (33.3%)	
	Type C	3 (14.3%)	2 (9.5%)	
Time before surgery (days)		1.9 ± 1.04	2.29 ± 1.34	0.311851
Operative time (minutes)		88.1 ± 16.9	124.05 ± 19.5	*P* < 0.001
Blood loss (cc)		84.29 ± 16.8	134.05 ± 31.2	*P* < 0.001
Follow-up period (months)		28.76 ± 6.04	31.1 ± 7.6	0.280379
Time of union (weeks)		15.48 ± 4.3	12.76 ± 3.7	0.035994

IMN: intramedullary nail; MIPO: minimal invasive plate osteosynthesis; N: number of patients in each group; RTA: road traffic accident. Sex, mechanism of injury, the affected side, and AO/OTA classification were compared by chi-square test, and the other variables were compared by independent *T*-test. *P* value less than 0.05 is considered significant.

**Table 2:** The final results of shoulder scores and complications for both groups.

	Group A	Group B	*P* value
ASES score	87.4 ± 14.5	95.01 ± 6.9	0.03
UCLA score	31.04 ± 4.4	33.3 ± 2.2	0.04
VAS	1.1 ± 1.08	0.54 ± 0.63	0.03
Complications number	5	2	0.214193
Nonunion	2	0	0.4878
Varus deformity	0	2	0.4878
Radial nerve injury	0	0	—
Infection	0	0	—
Shoulder complications	2 cases of subacromial bursitis; 1 case of partial rotator cuff tear	0	*P* < 0.001

ASES: American Shoulder and Elbow Surgeons; UCLA: University of California at Los Angeles Shoulder, and VAS; visual analog score. They were compared by independent *T*-test, while the complications by chi-square test and Fisher's exact test.

## Data Availability

The data used to support the findings of this study are available from the corresponding author upon request.
